# Malaria Incidence and Prevalence Among Children Living in a Peri-Urban Area on the Coast of Benin, West Africa: A Longitudinal Study

**DOI:** 10.4269/ajtmh.2010.09-0611

**Published:** 2010-09

**Authors:** Alain Nahum, Annette Erhart, Ambroisine Mayé, Daniel Ahounou, Chantal van Overmeir, Joris Menten, Harry van Loen, Martin Akogbeto, Marc Coosemans, Achille Massougbodji, Umberto D'Alessandro

**Affiliations:** Centre de Recherches Entomologiques de Cotonou, Cotonou, Bénin; Prince Leopold Institute of Tropical Medicine, Antwerp, Belgium; Hôpital de Zone, Abomey-Calavi, Bénin; Laboratoire de Parasitologie, Faculté des Sciences de la Santé, Université Nationale du Bénin, Cotonou, Bénin

## Abstract

Clinical malaria incidence was determined over 18 months in a cohort of 553 children living in a peri-urban area near Cotonou. Three cross-sectional surveys were also carried out. Malaria incidence showed a marked seasonal distribution with two peaks: the first corresponding to the long rainy season, and the second corresponding to the overflowing of Lake Nokoue. The overall *Plasmodium falciparum* incidence rate was estimated at 84/1,000 person-months, and its prevalence was estimated at over 40% in the two first surveys and 68.9% in the third survey. Multivariate analysis showed that girls and people living in closed houses had a lower risk of clinical malaria. Bed net use was associated with a lower risk of malaria infection. Conversely, children of families owing a pirogue were at higher risk of clinical malaria. Considering the high pyrethroids resistance, indoor residual spraying with either a carbamate or an organophospate insecticide may have a major impact on the malaria burden.

## Introduction

Malaria transmission and consequently, the disease burden may vary widely, even within a small geographical area.^1^–^3^ In the last few decades, research has been able to define new tools and strategies for malaria control such as artemisinin-based combination therapies (ACT), long-lasting insecticide-treated bednets (LLIN), intermittent preventive treatment in pregnancy (IPTp), and intermittent preventive treatment in infancy (IPTi).^4^,^5^ A wide variety of risk factors, socio-economic,^1^,^6^–^9^ environmental^10^–^15^ including housing conditions,^16^–^18^ and others,^19^–^23^ for malaria infection and disease, mostly specific to the local context, have been identified. Therefore, the formulation of a national malaria-control strategy should take into account the local context, the variations in malaria epidemiology, and hence, the approaches to its control that may occur even at a small scale.

In Benin, malaria remains the first cause of attendance to the health centers, despite the control activities carried out by the National Malaria Control Program.^24^ The malaria burden is probably higher than estimated by available data, because most patients are treated outside the formal health sector (Nahum A and others, unpublished data). However, no recent data on the actual malaria burden and the related risk factors are available. In the late 1990s, clinical malaria in children < 3 years old living in some coastal villages represented 33% of all febrile episodes with two yearly peaks.^25^ In 1992, the peri-urban sector of Cotonou, the economic capital, was identified as hyper-endemic after a series of cross-sectional surveys in children.^26^ No extensive study on the malaria burden in Southern Benin was carried out until 2003–2004 when a cohort of children living in the peri-urban lagoon area around Cotonou was followed-up for several months with the aims of establishing the malaria seasonality patterns, quantifying its burden, and identifying local risk factors. Results are reported below.

## Materials and Methods

### Study area.

**Figure 1.**
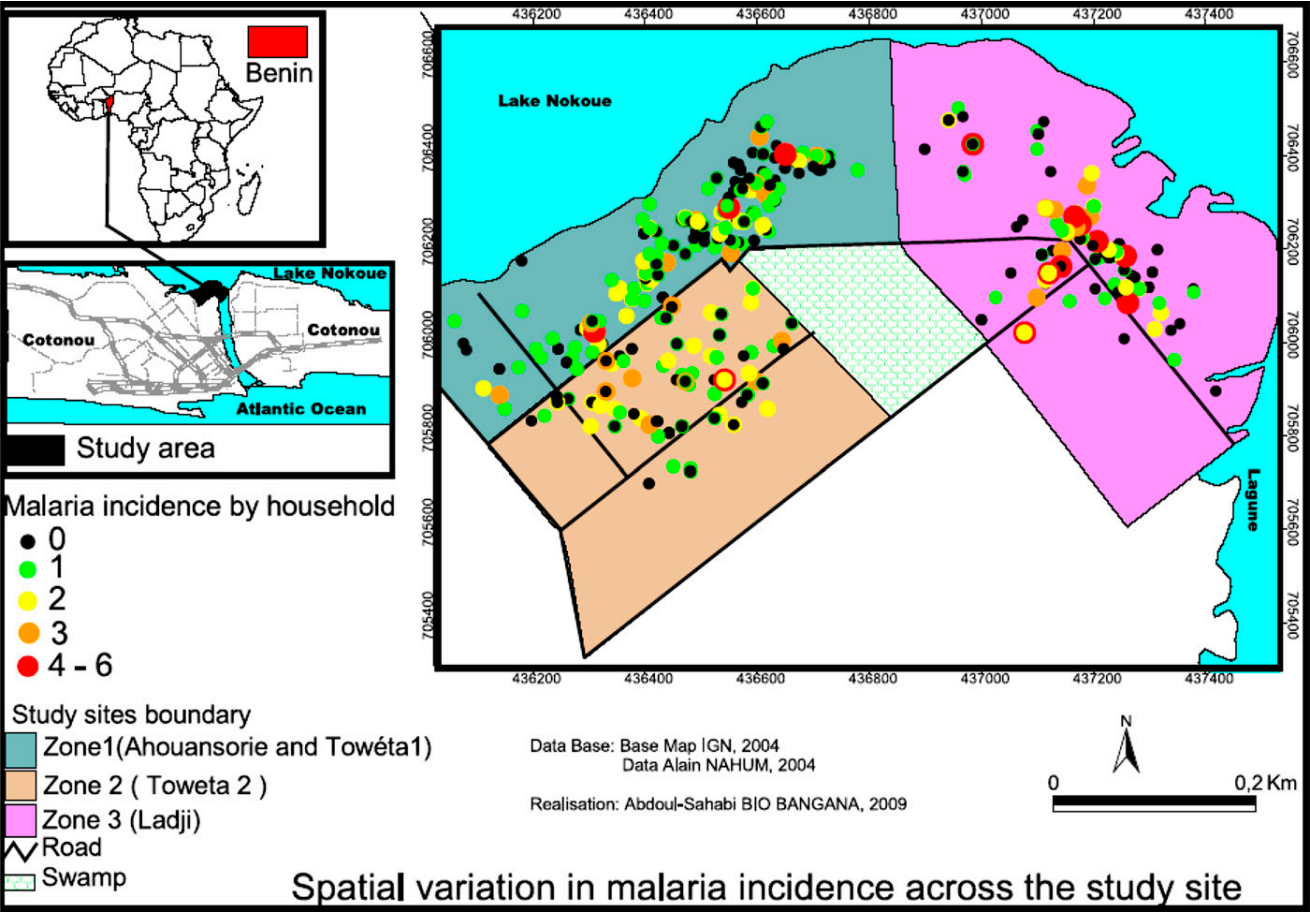
Study location in the coastal lagoon area near Cotonou, Republic of Benin, and spatial distribution of malaria cases by household. This figure appears in color at www.ajtmh.org.

The study was part of a longitudinal randomized trial carried out in southern Benin, West Africa, in three adjacent sites: Ladji, Awansori (Toweta 1), and Toweta 2, all located in a large suburban depression north of Cotonou bordering Lake Nokoue (Figure 1). The study area consists of a wide flat band (under sea level in some places) populated by migrants belonging to several ethnic groups from the surrounding regions living in poor houses with walls made of bamboo frames filled with either mud or cement and covered with corrugated iron roofs. Small trade represents the dominant economic activity.

The climate is subequatorial, with two rainy seasons (from April to July and from October to November) and two dry seasons (from December to March and from August to September). In 2003, monthly mean temperature varied between 23.6°C and 33.1°C, and the average relative humidity index was between 75.7% and 83.7%.^27^

During the long rainy season (from April to July), the environment does not change radically, with some fresh water pools mostly disappearing a few weeks after the end of the rains. In contrast, during the second season, this area is subject to recurrent flooding because of the overflowing of Lake Nokoue as a result of the water influx coming through the Oueme River, the main affluent of the lake, after the rains in Northern Benin.^26^

Malaria transmission, mainly by *Anopheles gambiae* s.s. although *An. melas* (4%) can also be found, is seasonal and lasts for about 8 months.^3^,^28^ The annual entomologic inoculation rate (AEIR) has been estimated at 58 infective bites per person.

### Study population.

In 2001, the local authorities and the population were informed about the study and its objectives. A census was carried out in 2002–2003. Houses were numbered, and information on the household's residents (name, sex, age, and relationship with the household head) were collected. A list of children 6–59 months old was produced according to the following criteria: absence of chronic illness with parents willing to participate in the study and unlikely to emigrate. Before inclusion, parents or legal guardians were asked to sign a written informed consent. Refusal to participate did not affect access to basic health services. Parents/guardians were instructed to attend the nearby health facility, where a physician was always available, whenever their child was sick. They were also asked to administer only drugs given or prescribed by the research physician to the child.

### Study design and procedure.

#### Household survey.

From February to March 2003, all participants' households were mapped using a handheld Global Position System (eTrex H personal navigator; Garmin) with an accuracy goal of < 15 m. If children lived in the same house or in an immediately adjacent one, only one point was recorded. A structured questionnaire combined to direct observation was used by a team of research assistants trained for this purpose to collect information on demographic variables, malaria recognition, treatment, and prevention as well as housing and environmental condition.

#### Cross-sectional surveys.

During the follow-up period, three cross-sectional surveys were carried out: the first from February to March 2003, the second from February to March 2004, and the third at the end of the flooding period between October and November 2004. During surveys, each child was examined by a physician, and a blood sample for hematology, the detection of malaria infection (microscopy), and later, genotyping was collected.

#### Longitudinal follow-up.

Follow-up of the cohort of children for the active detection of clinical malaria cases started in July 2003 and ended in December 2004. During this period, children were visited at home two times per week. At each visit, auxiliary temperature was checked, and if found ≥ 37.5°C, a blood sample for microscopic examination (thick and thin blood film) and later, genotyping (Whatman filter paper grade 3) was collected. Similar procedures were carried out when cohort children attended the health facilities within the study area. Clinical malaria was defined as fever (axillary temperature ≥ 37.5°C) with a positive blood slide for *Plasmodium falciparum* asexual stages, regardless of parasite density. Children with a *P. falciparum* monoinfection, a parasite density between 1,000–200,000/μL, and without the following exclusion criteria [packed cell volume (PCV) < 15%, severe malaria,^29^ danger signs (prostration, inability to drink, recent convulsion, persistent vomiting), other concomitant illness, or underlying disease] were treated with chloroquine (CQ; 25 mg/kg over 3 days), sulfadoxine-pyrimethamine (SP; 25 mg/kg of sulphadoxine and 1.25 mg/kg of pyrimethamine in a single dose), or SP and artesunate (SP-AS; SP single dose and AS dose of 12 mg/kg over 3 days) according to a predefined randomization list; active follow-up occurred until day 28 post-treatment.^30^ Children belonging to a treatment group received the same treatment as other uncomplicated malaria episodes identified during the whole surveillance period. Severe malaria cases were treated with quinine as recommended by the National Malaria Control Program.^31^ Patients with uncomplicated malaria but not meeting the inclusion criteria (parasite density < 1000/μL and mixed infections) were treated according to national treatment policy at the time of the study [i.e., CQ (25 mg/kg over 3 days)].^31^ Other illnesses were treated accordingly; patients were referred to the hospital when needed.

### Laboratory methods.

#### Parasite count and hematological assays.

Thin blood films were fixed with methanol and stained, together with thick films, with Giemsa 10% for 10 minutes. Parasite density was determined according to the number of parasites per 200 white blood cells (WBC), assuming a total WBC count of 8,000/μL. If gametocytes were seen, the gametocyte count was extended to 1,000 WBC. Slide reading was blinded to patients' identity and treatment allocation. Packed cell volume was measured in microcapillary tubes using a Hawkesley hematocrit reader after centrifugation.

#### Parasite genotyping.

Parasite genotyping to distinguish between a new infection and a recrudescence was done on samples collected during two consecutive malaria episodes when the second one occurred at least 14 days after the first episode. *P. falciparum* DNA was purified, and genotyping was done by nested polymerase chain reaction (PCR) for variable blocks within the merozoite surface protein 1 (MSP1) and 2 (MSP2) as described previously.^32^,^33^ A recrudescence was defined when at least one common band was observed for both markers in the day 0 sample and at the day of recurrent parasitemia. A new infection was defined when at least one of the two markers had a completely different pattern between day 0 and the day of recurrent parasitemia.

### Sample size.

The sample size was calculated on the basis of the previous estimation of CQ treatment failure (20%) in southern Benin and the assumption that SP and SP-AS treatment failure would not be higher than 5%.^34^ Eighty-eight children per treatment would be needed to detect, at 80% power and 5% significance level, a significant difference between CQ and the other two treatments. It was also assumed that the incidence of clinical malaria would be 0.8 per year^25^ and that about one-half of clinical cases would be either not eligible or lost to follow-up, resulting in a target sample size of 553 children.

### Statistical analysis.

Data were analyzed with STATA version 10 (Stata Corp., College Station, TX). Descriptive statistics were used to summarize baseline values and demography data.

Possible predictors of clinical malaria (PCR-corrected) and infections at cross-sectional surveys were studied using logistic regression models. Closed houses were defined as those without eaves, with a ceiling, and with the possibility of closing windows and doors. To correct for dependencies between repeated measures on the same child, robust standard errors were used with child as the clustering unit and either the weeks in observation for clinical malaria or the survey number for the parasitemia as the time unit. The association between possible predictors and malaria was assessed by using bivariate logistic regression models. Factors with *P* value < 0.200 were selected for inclusion in a multivariate model. The multivariate model was constructed using a backwards elimination procedure that retained only factors with a *P* value < 0.05. Significance was assessed using Wald tests at the 5% two-sided significance level. The map showing the spatial distribution of malaria cases by households was produced using Arc View software 3.2. Spatial clustering of malaria cases, compared with children without malaria, was tested using the *k*-nearest neighbor method.^35^ The *P* values for the different values of *k* were calculated through the Monte Carlo simulation, with ties between children in the same household randomly broken.

### Ethical approval.

The study was approved by the Minister of Health of Benin, the Ethical Committee of the Faculté des Sciences de la Santé, Cotonou, Benin, and the Ethical Committee of the Institute of Tropical Medicine, Antwerp, Belgium. Informed consent was obtained from parents or guardians.

## Results

### Socio-demographical characteristics of the study population.

**Figure 2.**
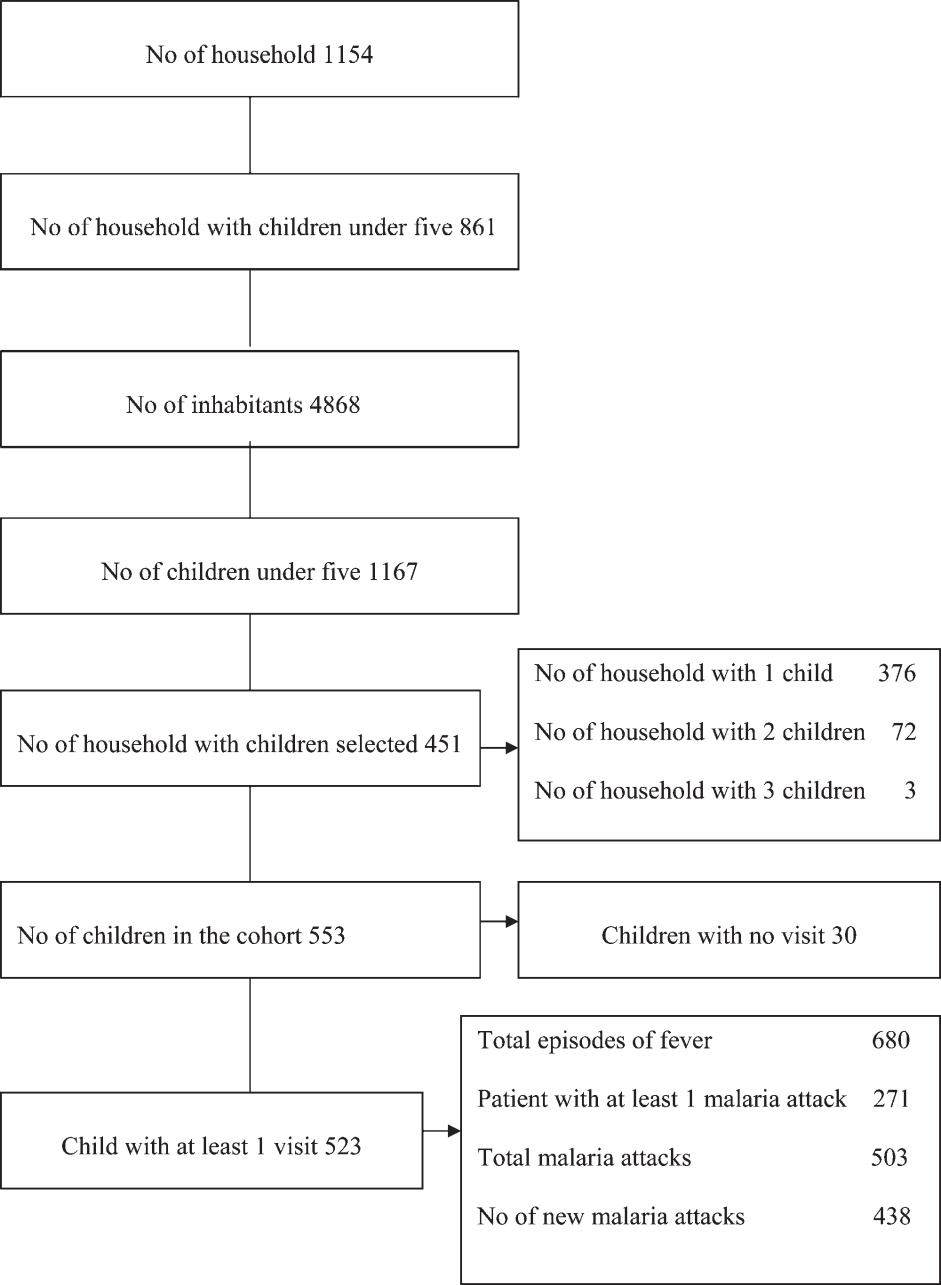
Census characteristics and clinical malaria episodes diagnosed during the follow-up. No = number.

At the beginning of the study, the census identified 1,167 children < 5 years old distributed in 861 households; 553 (47.39%) of them were randomly selected to be included in the cohort under surveillance (Figure 2), and most of them (94.58%; 523) had at least one home visit during the 78 weeks under surveillance. However, the mean number of observation-weeks was 48 [interquartile range (IQR) = 28–57) for a total of 5,238 person-months of the possible 9,414 person-months (Table 1). Missed visits were because of the difficulties of doing home visits during the flooding and the temporary absence of people. About one-half of the children lived in Toweta 1, which is located, similarly to Ladji, along the lake shores. Bed net use among the cohort children was 54.8% (Table 1). About one-half of the mothers were illiterate, most of them aged > 30 years, with irregular income and coming from outside the study area (Table 1). Few caregivers had an economic activity related to fishing. Most houses were build with bricks, and only 7% (37/528) could be defined as “closed houses” (Table 1).

### Clinical malaria episodes.

**Figure 3.**
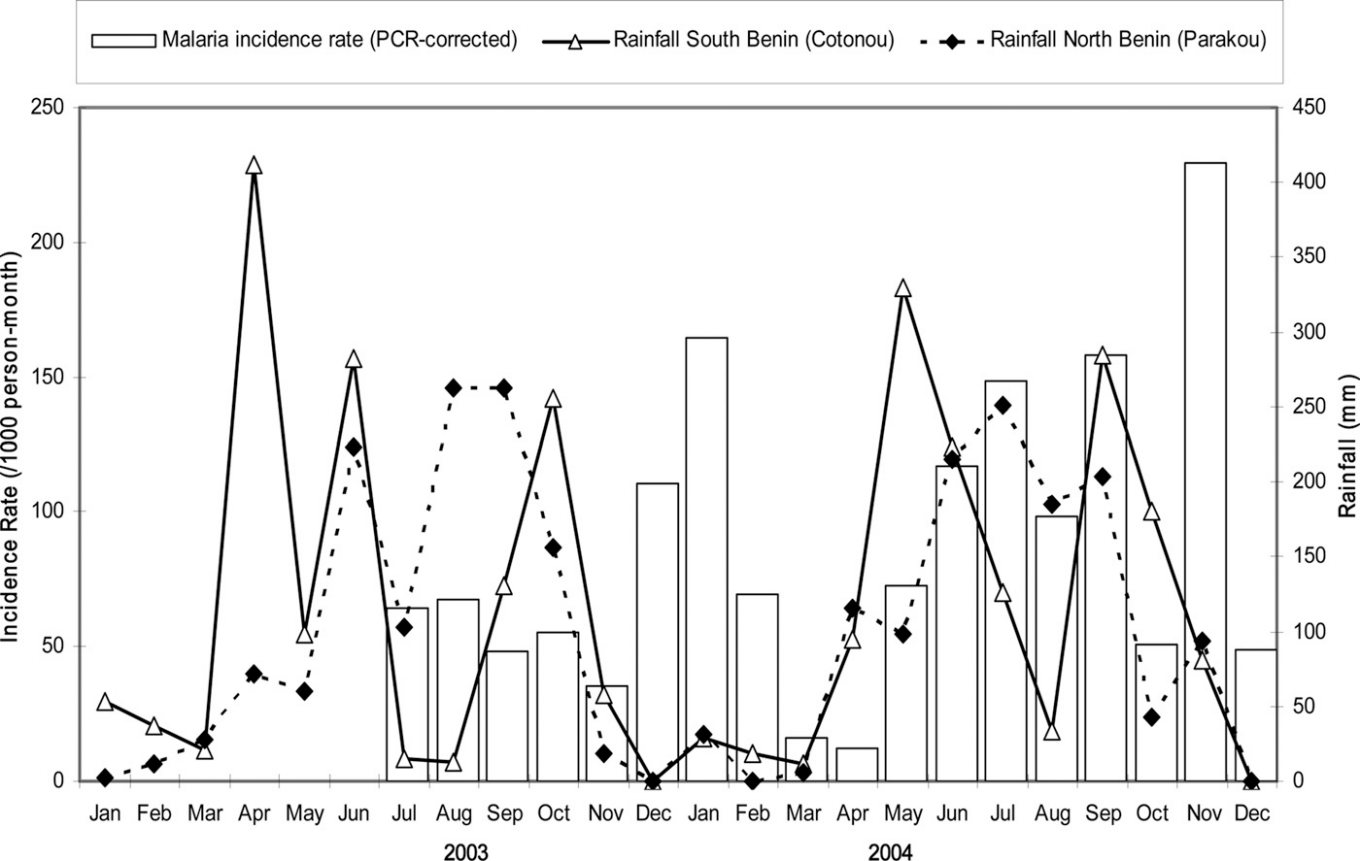
Monthly clinical malaria incidence rate during the study period. Two years of follow-up were combined.

During the surveillance period, 680 episodes of fever were detected, among which 503 were diagnosed as *P. falciparum* clinical malaria episodes in 271 (51.8%) children. In total, 438 new infections, not evenly distributed, were identified: 160 children had just one malaria episode, whereas 111 had two or more episodes (71 children had two episodes, 28 children had three, 10 children had four, and 2 children had six). No death occurred in the cohort during the surveillance period. Among the 438 episodes of clinical malaria detected during the follow-up, 202 (46.1%) fulfilled the inclusion/exclusion criteria and were treated with CQ, SP, or SP-AS. Malaria incidence showed a marked seasonal distribution (Figure 3), with an overall incidence rate estimated at 84/1,000 person-months and large differences between months, varying from 12 to 230 per 1,000 person-months (Table 2). The months of lowest incidence were March and April 2004, with an incidence estimated at 16 and 12/1,000 person-months, respectively, corresponding to the end of the long dry season. Both in 2003 and 2004, the incidence showed two peaks: the smaller peak from July to September and the bigger peak from November to January. The former corresponds to the long rainy season, and the latter corresponds to the overflowing of Lake Nokoue (Figure 3). The incidence in 2004 was significantly higher than in 2003 [e.g., in July, the risk of clinical malaria was more than two times higher than in the same month in 2003; odds ratio (OR) = 2.35; 95% confidence interval (CI) = 1.38–3.99; *P* = 0.002) (Table 2). Such increase was not linked to any obvious ecological or environmental change. Households in which children did not have any clinical attack during the surveillance period were interspersed with those in which one or more clinical attacks were observed (Figure 1). No specific clustering of clinical cases could be observed (*P* = 0.256; *k* = 5).

In the univariate analysis at a *P* value < 0.200, several risk factors were associated with the risk of clinical malaria (i.e., gender, locality, housing conditions, type of bed and bed net use, ownership of a pirogue, ownership of a fishing net) and considered for the multivariate modeling (Table 3). Because the two later variables were strongly correlated and could not both be included in the final multivariate model, we choose pirogue rather than fishing net, because the former may be filled with water and become a vector-breeding site when ashore and not used. In the multivariate analysis, girls seemed to have a lower risk of clinical malaria (OR = 0.82; 95% CI = 0.67–1.0; *P* = 0.04); similarly, people living in closed houses also had a lower malaria risk (OR = 0.65; 95% CI = 0.42–0.99; *P* = 0.04). Conversely, children of families owing a pirogue were at higher risk of clinical malaria (OR = 1.53; 95% CI = 1.16–2.03; *P* = 0.003) (Table 3).

### Cross-sectional surveys and malaria infection.

Participation to the cross-sectional surveys decreased over time but always remained over 80% (Table 4). The prevalence of *P. falciparum* infection was high in all surveys (over 40%), with no difference between the first two surveys and a significantly higher prevalence in the third survey (68.9%; *P* < 0.001) carried out in October–November 2004 (Table 4). The proportion of children identified at the time of the survey as having a clinical attack according to the definition given above decreased from the first to the third survey; it was significantly higher at the first survey (22.2%) compared with the second (9.2%; *P* < 0.001) and third surveys (14.2%; *P* = 0.005) (Table 4). Similarly, the mean parasite density decreased steadily between survey 1 and 3. Gametocyte prevalence was around 10% at each survey. The spleen rate was also high (the great majority belonging to the Hackett's class 2 or more), with survey 2 having a significantly lower value (39.09%; *P* = 0.008) (Table 4).

The risk of malaria infection was significantly higher at survey 3, which was at the time of flooding (OR = 3.01; 95% CI = 2.19–4.15; *P* < 0.001) (Table 5) and for children living in Toweta 2 (OR = 1.41; 95% CI = 1.08–1.86; *P* < 0.012). There was a statistically significant interaction between surveys and localities. However, the time trends were similar across sites and the interaction seemed of little epidemiological relevance; therefore, we present results pooled over the three sites. Regular bed net use was associated with a significantly lower risk of infection (OR = 0.77; 95% CI = 0.61–0.97; *P* < 0.026).

In surveys 2 and 3, there was no difference in malariometric indices between children having received antimalarial treatment and those who had not; similarly, there was no difference according to the treatment received (data not shown).

## Discussion

In this peri-urban lagoon area near Cotonou, malaria transmission is perennial with the parasite, and the spleen rates are between 40% and 60%, indicating that this is a meso- to hyperendemic area where malaria infection and disease represent a substantial burden for the local population. Ten years earlier, the malaria prevalence was higher, constantly over 75%,^26^ with a high prevalence in the 5- to 9-year-old group. However, a more recent study carried out in Cotonou in March 2003 reported an overall prevalence of infection of 5.2% among primary school children, with some variability between center and periphery but with values always lower than 10%.^36^ This was part of an extensive study on the burden of malaria in urban areas located in sub-Saharan Africa^37^ [i.e., the Rapid Urban Malaria Appraisal (RUMA) that, besides Cotonou, investigated urban malaria in Dar es Salaam,^38^ Abidjan,^39^ and Ouagadougou].^40^ In Cotonou, the prevalence of malaria infection among febrile cases detected at health facilities was extremely low, 0% in infants and only 6.8% in children 1–5 years (1.4% and 2.8% in non-febrile controls), with little difference between the center and the periphery of the town; however, 34% of the consultations in health facilities had a presumptive (without microscopy) diagnosis of clinical malaria and were treated accordingly.^36^ The difference in malaria prevalence between the RUMA's and the cohort's surveys is particularly striking when considering that both were done at about the same period. Nevertheless, Ménontin, the health center in the periphery of Cotonou where the prevalence of infection among febrile cases was determined by RUMA, is close to Toweta 1 and 2 and Ladji, where the cohort children lived. It is, therefore, surprising that, in the RUMA's study, the percentage of true malaria cases among the patients diagnosed at the health center was extremely low, suggesting that the great majority of malaria patients was treated at home.^36^ Conversely, antimalarial treatment was administered presumptively to about one-third of patients,^36^ creating the paradoxical situation in which patients attending health facilities received treatment that would have been mostly useful to those not attending. It is unclear whether attendance to health facilities and diagnosis has improved since the change in 2004 of the first-line treatment for uncomplicated malaria from CQ to either artemether–lumefantrine or amodiaquine–artesunate.^24^ The deployment of these ACTs has been slow, although this is gradually improving.^41^ However, the availability of ACT at peripheral health facilities, although essential, may not be able to decrease the malaria burden if patients do not attend and the diagnosis is not correctly done. It is well-known that most malaria (or febrile) episodes are first treated at home using shop-bought drugs,^42^ and this mostly occurs, because malaria is considered as a simple disease.^43^ The widespread use of antimalarial drugs outside the formal health sector is the basis for promoting the home-based management of malaria cases (HMM). However, the current evidence showing the health benefit of home- and community-based presumptive treatment of fever with antimalarials is limited, and it does not necessarily support its widespread implementation, particularly regarding the use of ACTs for this purpose.^44^ Nevertheless, in the study-area setting, HMM may be an option for increasing the access to adequate antimalarial treatment, although the issue of correct diagnosis remains, because this is usually done presumptively within the HMM.

This is the first longitudinal community-based study on malaria morbidity carried out in Benin with more than 12 months of follow-up. Clinical episodes were identified by weekly active case detection. This methodology has been used in several other settings in sub-Saharan Africa and is obviously superior to monthly visits, which, considering the widespread practice of self-medication, may detect only a proportion of all cases.^15^,^45^,^46^ Because of the continuous presence of field workers, about one-third of visits were missed, mostly because of the mother's trade activities, attendance to traditional ceremonies, or difficult access because of the floods, illustrating the difficulty of carrying out these studies.

Although no specific clustering of clinical cases could be observed, children living in Toweta 2 had a higher risk of clinical malaria than those in Toweta 1 and Ladji, both on the lake shores, possibly because of the higher number of permanent or temporary mosquito breeding sites, like swamps or freshwater pools. These probably increase at the time of annual floods,^26^ determining the second annual peak of malaria incidence in November–January.

The occurrence of clinical malaria was not evenly distributed among all the cohort children, and only one-half of them had one or more clinical episodes over the 18-month period. This proportion is low but consistent with previous findings from others studies in Africa.^6^,^9^

The absence of clustering of malaria cases suggests the presence of factors other than host genetic polymorphisms [e.g., the protective effect of sickle cell trait and the glucose-6-phosphate dehydrogenase (G-6-PD) deficiency, which is showed to be associated with clinical malaria heterogeneity].^47^–^50^ Possibly, housing construction and environmental conditions could influence the risk of infection.^47^–^49^

The apparent difference in the last survey (October–November 2004) between the relatively low number of children with clinical malaria and the high prevalence of infection may be explained by the active detection of clinical cases (two times per week) and their prompt treatment.

Use of bed nets was relatively common, although most of them were probably not treated with insecticide. The protective effect of both treated and untreated mosquito nets against malaria infection is well-documented.^51^–^53^ Nevertheless, high mosquito resistance to pyrethroids in the study area has been recently reported.^54^ The loss of insecticidal effect was calculated to be > 95%, although insecticide-treated bed nets could partially deter mosquitoes entering houses. Despite such high resistance, bed net use in this cohort of children significantly decreased the risk of both clinical malaria and malaria infection, possibly because of the mechanical barrier against mosquito bites (only a small percentage of them had been treated with insecticide).^55^ It is unclear whether the distribution of insecticide-treated bed nets or even indoor residual spraying in this area would substantially decrease the malaria morbidity. Probably, an insecticide other than a pyrethroid, such as a carbamate or an organophosphate, should be used. This should be done only after careful assessment of the insecticide resistance of the local vector population.^56^

In the early 20th century, improved housing and screening were considered as valid methods for controlling malaria.^57^ Decreased house entry by *An. gambiae*, the principal African malaria vector, with ceiling and closed eaves has been recently reported.^58^ Therefore, it is not surprising that children living in closed houses, defined as those without eaves, with a ceiling, and with the possibility of closing windows and doors, should have a significantly lower risk of clinical malaria. Such association is likely to be confounded by the socio-economic status, because this type of house was uncommon and probably inhabited by wealthier people. Nevertheless, the majority of houses did not have any protection against mosquito entry, whereas the closed houses offered some mechanical barrier to the vector.

Few families owned a pirogue, but this was a strong risk factor for clinical malaria. In this impoverished community, mostly composed by immigrants, fishing has ceased to be a major economic activity, and pirogues are not used anymore but left outside the houses. Nevertheless, unused pirogues probably play a role as a breeding site after being filled with water during the rainy season. This should be an easily solvable problem, although unlikely to have a major impact on transmission.

In conclusion, malaria transmission in this peri-urban area is substantial and perennial, with two peaks: the first corresponding to the long rainy season, and the second corresponding to the overflowing of Lake Nokoue caused by the increased water flow after the rains in Northern Benin. Considering the high resistance to pyrethroids and the high population density, indoor residual spraying with either a carbamate or an organophospate insecticide may have a major impact on the malaria burden. Improvement of the population's socio-economic status and housing conditions could reduce it further.

## Figures and Tables

**Table 1:** Baseline characteristics

	No	%
No. of weeks observed		
0	30	5.4
1–19	75	13.6
20–39	93	16.8
40–59	259	46.8
> 60	96	17.4
Age (month)		
≤ 24	171	30.9
25–36	131	23.7
37–48	108	19.5
49–59	143	25.9
Male/Female	283/270	51.2/48.8
Site		
Toweta 1	291	52.6
Toweta 2	125	22.6
Ladji	137	24.8
Bed net use*	290	54.8
Type of bed†		
Mat	228	43.2
Bed without mattress or mattress alone	300	56.8
Caregiver*		
Mother	457	86.4
Others	72	13.6
Caregiver's age (years)*		
< 25	139	26.3
26–30	185	35.0
> 30	205	38.8
Caregiver's education level*		
Illiterate	268	50.7
Primary/secondary/high level	203	49.3
Caregiver's occupation*		
Foods seller	179	33.8
Various article seller	233	44.1
Handy profession	54	10.2
Housewife/nothing	23	4.4
Trader	20	3.8
Employees	20	3.8
Ethnic group†		
Xwla or Toffin (local)	184	34.9
Fon-Goun Aïzo Setto torri Houeda	233	44.1
Mina Adja Pedah Watchi Kotafon sahoue	63	11.9
Mahi Yoruba Idatcha Nago	48	9.1
Owning a pirogue*	32	6.1
Owning a fishing net*	45	8.5
House†		
Bamboo or bamboo and brick	103	19.5
Brick	425	80.5
Closed house	37	7.0

No. = number.

* Data missed for 24 children.

† Data missed for 25 children.

**Table 2:** Clinical malaria (new infection) and month as risk factor

Month	Cases/PM	Incidence rate/1,000 PM	OR (95% CI)	*P*	Adjusted OR	*P*
2003						
July	23/360.0	64	1	–	1	
August	24/356.4	67	1.05 (0.59–1.88)	0.8	1.07 (0.60–1.91)	0.8
September	17/352.8	48	0.75 (0.40–1.39)	0.4	0.76 (0.41–1.41)	0.4
October	19/344.4	55	0.86 (0.47–1.57)	0.6	0.88 (0.48–1.61)	0.6
November	12/337.2	36	0.55 (0.29–1.07)	0.08	0.56 (0.29–1.08)	0.08
December	39/352.8	111	1.75 (1.07–2.85)	0.02	1.77 (1.08–2.89)	0.02
2004						
January	62/376.8	165	2.64 (1.64–4.25)	> 0.001	2.67 (1.66–4.31)	> 0.001
February	14/201.6	69	1.09 (0.57–2.09)	0.8	1.01 (0.52–1.97)	0.97
March	5/307.2	16	0.25 (0.09–0.67)	0.005	0.26 (0.1–0.68)	0.006
April	3/252	12	0.18 (0.05–0.61)	0.006	0.18 (0.05–0.61)	0.006
May	19/261.6	73	1.14 (0.62–2.08)	0.7	1.15 (0.63–2.1)	0.6
June	33/283.2	117	1.85 (1.12–3.06)	0.02	1.86 (1.12–3.09)	0.02
July	28/188.4	149	2.37 (1.40–4.01)	0.001	2.35 (1.38–3.99)	0.002
August	19/194.4	98	1.54 (0.86–2.77)	0.14	1.55 (0.87–2.78)	0.14
September	45/285.6	158	2.52 (1.51–4.22)	> 0.001	2.54 (1.52–4.24)	> 0.001
October	16/316.8	51	0.79 (0.42–1.48)	0.46	0.79 (0.42–1.49)	0.47
November	47/205.2	229	3.73 (2.29–6.08)	> 0.001	3.75 (2.30–6.11)	> 0.001
December	13/265.2	49	0.77 (0.39–1.48)	0.43	0.77 (0.40–1.49)	0.44
Total	438/5,238	84				

PM = person-month; OR = odds ratio; CI = confidence interval; *P* = *P* value.

**Table 3:** Clinical malaria (new infection) and related risk factors: multivariate adjusted analysis using logistic regression

Risk factors	Cases/PM	Incidence rate/1,000 PM	OR (95% CI)	*P*	Adjusted OR*	*P*
Month	438/5,238.0	84	Table 2	< 0.001	Table 2	< 0.001
Sex						
Boys	245/2,707.2	91	1	–		
Girls	193/2,532.0	76	0.84 (0.69–1.02)	0.084	0.82 (0.67−1.0)	0.046
Site						
Toweta 1	201/2,586.0	78	1	–		
Toweta 2	105/1,310.4	80	1.03 (0.82–1.30)	0.8		
Ladji	132/1,341.6	98	1.27 (1.00–1.62)	0.054		
Closed house						
No	418/4,850.4	86	1	–	1	–
Yes	19/354.0	54	0.62 (0.40–0.94)	0.026	0.65 (0.42–0.99)	0.047
Type of bed						
No bed	211/2,272.8	93	1	–		
Bed	226/2,931.6	77	0.83 (0.68–1.01)	0.059		
Bed net use						
No	223/2,356.8	95	1	–		
Yes	214/2,846.4	75	0.79 (0.65–0.96)	0.019		
Pirogue						
No	383/4,778.4	80	1	–	1	–
Yes	54/424.8	127	1.60 (1.22–2.10)	0.001	1.53 (1.16–2.03)	0.003
Fishing net						
No	372/4,663.2	80	1	–		
Yes	65/541.2	120	1.52 (1.18–1.95)	0.001		

No. = number; PM = person-month; OR = odds ratio; CI = confidence interval; *P* = *P* value.

* Only statistically significant adjusted ORs were reported.

**Table 4:** Malaria prevalence and other malariometric indices by survey

	February–March 2003	February–March 2004	October–November 2004
N (% children cohort)	500 (90.4)	381 (80.6)	338 (85.4)
*P. falciparum* infection (%)	203 (40.6)	155 (40.7)	233 (68.9)
*P. falciparum* clinical malaria (%)	111 (22.2)	35 (9.2)	48 (14.2)
Mean (geometric) *P. falciparum* density (IQR)	276 (210–364)	174 (117–260)	88 (67–118)
Gametocyte carriers (%)	52 (10.4)	33 (8.7)	28 (8.5)
Mean PCV (IQR)	37.4 (37.0–37.7)	37.2 (36.8–37.6)	36.6 (36.2–37.0)
Enlarged spleen (%)	230/475 (48.4)	138/353 (39.1)	141/283 (49.8)
Hackett Class 1 (%)	32 (13.9)	25 (18.1)	20 (14.2)
Hackett Class > 1 (%)	198 (86.1)	113 (81.9)	121 (85.8)

*N* = total number of children in the cohort at the time of survey; IQR = interquartile range; PCV = packed cell volume.

**Table 5:** Malaria infection and related risk factors

Risk factors	Cases (*n*/*N*)	Prevalence	OR (95% CI)	*P*	Adjusted OR* (95% CI)	*P*
Survey	591/1,219	48.5				
Survey_1	203/500	40.6	1	–	1	
Survey_2	155/381	40.7	1.00 (0.76–1.32)	1.0	0.93 (0.70–1.23)	0.6
Survey_3	233/338	68.9	3.25 (2.41–4.37)	< 0.001	3.01 (2.19–4.15)	< 0.001
Age (months)	591/1,219	48.5				
≤ 24	81/236	34.3	1		1	
25–36	144/274	52.6	1.73 (1.20–2.50)	0.004	1.74 (1.20–2.53)	0.004
37–48	119/236	50.4	1.48 (1.01–2.18)	0.04	1.53 (1.04–2.27)	0.03
> 49	247/473	52.2	1.38 (0.99–1.93)	0.05	1.36 (0.98–1.90)	0.07
Site	591/1,219	48.5				
Toweta 1	299/637	46.9	1		1	
Toweta 2	157/291	54.0	1.37 (1.05–1.80)	0.02	1.41 (1.08–1.86)	0.01
Ladji	135/291	46.4	0.99 (0.74–1.32)	0.92	1.00 (0.75–1.35)	0.98
Type of bed	585/1,208	48.4				
Mat	269/530	50.8	1			
Bed without mattress or mattress alone	316/678	46.6	0.82 (0.65–1.04)	0.11		
Bed net use	585/1,208	48.4				
No	288/559	51.5	1		1	
Yes	297/649	45.8	0.79 (0.63–1.00)	0.05	0.77 (0.61–0.97)	0.03

*n* = number of clinical malaria cases; *N* = total number of children surveyed; OR = odds ratio; CI = confidence interval; *P* = *P* value.

* Only statistically significant adjusted ORs were reported.
